# Advancements in Rectal Drug Delivery Systems: Clinical Trials, and Patents Perspective

**DOI:** 10.3390/pharmaceutics14102210

**Published:** 2022-10-17

**Authors:** Ritu Rathi, Sanshita, Alpesh Kumar, Vivekanand Vishvakarma, Kampanart Huanbutta, Inderbir Singh, Tanikan Sangnim

**Affiliations:** 1 Chitkara College of Pharmacy, Chitkara University, Rajpura 140401, India; 2 School of Pharmacy, Eastern Asia University, Pathumthani 12110, Thailand; 3 Faculty of Pharmaceutical Sciences, Burapha University, Chonburi 20131, Thailand

**Keywords:** rectal, rectal diseases, rectal drug delivery, novel drug delivery, clinical trials

## Abstract

The rectal route is an effective route for the local and systemic delivery of active pharmaceutical ingredients. The environment of the rectum is relatively constant with low enzymatic activity and is favorable for drugs having poor oral absorption, extensive first-pass metabolism, gastric irritation, stability issues in the gastric environment, localized activity, and for drugs that cannot be administered by other routes. The present review addresses the rectal physiology, rectal diseases, and pharmaceutical factors influencing rectal delivery of drugs and discusses different rectal drug delivery systems including suppositories, suspensions, microspheres, nanoparticles, liposomes, tablets, and hydrogels. Clinical trials on various rectal drug delivery systems are presented in tabular form. Applications of different novel drug delivery carriers viz. nanoparticles, liposomes, solid lipid nanoparticles, microspheres, transferosomes, nano-niosomes, and nanomicelles have been discussed and demonstrated for their potential use in rectal administration. Various opportunities and challenges for rectal delivery including recent advancements and patented formulations for rectal drug delivery have also been included.

## 1. Introduction

The rectum represents a chamber present at the end of the large intestine in which drugs can be easily administered and can be well absorbed. Rectal administration is a secondary choice after oral and intravenous (IV) routes of drug administration and offers various advantages such as retention of large volumes, instant absorption of low molecular weight drugs, by-passing of the first-pass metabolism, controlled drug delivery, absorption into the lymphatic system, improved efficacy of localized treatment, enhanced absorption, and helps to administer gastric unstable drugs. The rectal route becomes the first choice in certain conditions like nausea, vomiting, objectionable taste, unconsciousness during post-operative treatments, and difficulty in swallowing, for patients with motility issues in the gastric tract like dysphagia, or if there is an inflammation at the site of intra-muscular administration. The rectal drug delivery system has been neglected due to some barriers such as erratic absorption, dissolution problems due to the small fluid content of the rectum, limited absorption surface area, drug metabolism, privacy concerns, and poor patient compliance [1]. This review addresses the physiological considerations of rectal drug delivery for treating different types of rectal diseases. Conventional and novel rectal drug delivery approaches have also been discussed systematically. The later part of the review mentions clinical trials, patented products, and various challenges associated with rectal drug delivery systems.

The rectum is located at the end of the large intestine and terminates at the anus serving as a temporary storage area for the defecation process. In an adult, the rectum is about 15–20 cm long, and 15 to 30 cm in diameter; the fluid volume is 1–3 mL having a pH of 7.2–7.4. The shape of the rectum may be pear-like, balloon-like, or tube-like and its size is larger in men as compared to women.

**Figure 1:**
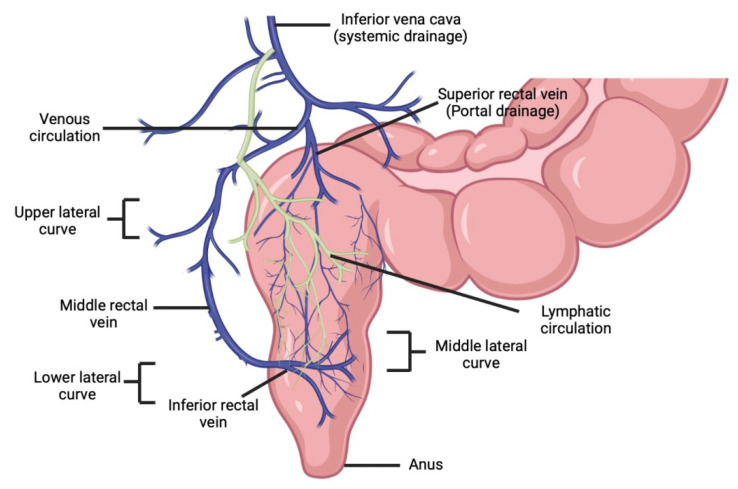
Schematic showing venous and lymphatic drainage from the rectum and portosystemic shunting.

The rectum is made up of columnar epithelial cells with numerous goblet cells, which are responsible for mucus secretion. In comparison with the small intestine, the rectum has a smaller surface area of about 200 to 4000 cm^2^ because of the absence of villi and microvilli on the luminal surface of the rectum. Rectal drainage is controlled by three veins, namely the superior, middle, and inferior rectal veins. The superior rectal vein drains the upper part of the rectum (via the inferior mesenteric vein) into the portal venous system; the middle and inferior rectal vein drains the lower part of the rectum into the internal iliac vein (via the internal pudendal vein) for systemic circulation as depicted in Figure 1 [2,3].

The rectum has a much lower surface area but is potentially beneficial for the drugs that:

i. have poor gastrointestinal absorption
ii. have low solubility, stability, and permeability
iii. undergo extensive first-pass metabolism
iv. cause irritation to the gastric mucosa
v. are unstable or degradable in the gastrointestinal tract
vi. have localized action in the rectum
vii. could not be administered by any other route [4].

## 2. Factors Influencing Rectal Drug Delivery

**Figure 2:**
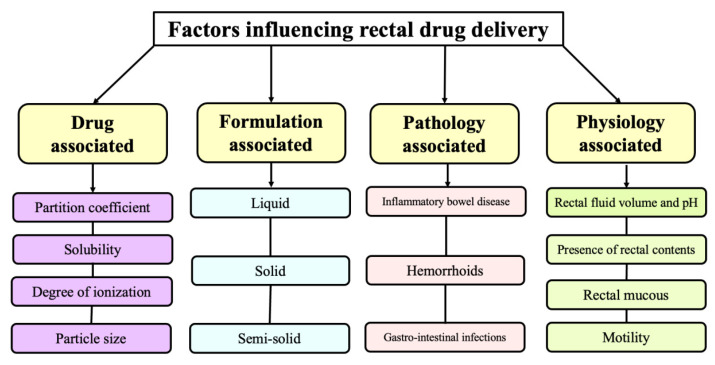
Factors influencing rectal drug delivery.

There are various factors which can affect rectal drug delivery and can be broadly classified into four categories, as depicted in Figure 2.

### 2.1. Drug Associated Factors

#### 2.1.1. Partition Coefficient

The value of logP is a measure of the lipophilicity or hydrophobicity of the drug molecule. There are two routes for the absorption of drugs in the rectum: transcellular (major route) and paracellular. The lipophilicity of the drug has an impact on how well it can pass through the rectal epithelium, and it affects the absorption of drug through transcellular route proportionally. The drug absorbs more readily when its lipophilicity is higher. However, for effective rectal drug administration, it is preferable to have a balance between lipophilicity and hydrophilicity. Drugs must be sufficiently lipophilic to pass the epithelium and enough hydrophilic to dissolve in rectal fluid [5].

#### 2.1.2. Solubility

Before the drug passes through the mucus layer and epithelium, it must first become solubilized in the rectal fluid. The value of solubility will affect the concentration of the drug available for absorption through the rectal route. Higher solubility favors higher dissolution and hence faster absorption [6].

#### 2.1.3. Dissociation Constant and Degree of Ionization

The existence of a drug in its ionized or unionized form is another factor affecting drug absorption through the rectal route. Unionized drugs are more lipophilic as compared to ionized drugs and show higher absorption through the transcellular route. Basic drugs (with the dissociation constant pKa near or above the physiologic range) exist more in the unionized form at the physiological pH of the rectum and show higher absorption.

#### 2.1.4. Particle Size

Another factor that influences rectal drug delivery is the particle size of the drug. The smaller the size, the faster the dissolution and absorption. Small particles have a large surface area to volume ratio which leads to a higher dissolution rate and solubility, therefore faster absorption. Drugs with a particle size of range 50–100 µm show the maximum absorption through the rectal route.

### 2.2. Formulation Associated Factors

The type of formulation of rectal dosage form used also influences the absorption of the drug.

#### 2.2.1. Liquid Formulations

The drug release from formulation and its solubilization in the rectal fluid is very fast in liquid formulations. It has also been seen that liquid formulations have a greater spreading capacity and help to provide local and systemic benefits of the drug.

#### 2.2.2. Solid Formulations

Solid dosage forms administered rectally undergo disintegration, liquefaction, and dissolution for drug release before the drug can be absorbed and can cross the epithelium. Therefore, the time taken to obtain a therapeutic effect is higher for solid formulations than for liquids.

#### 2.2.3. Semi-Solid Dosage Forms

In order to treat local conditions of ano-rectal pruritus, inflammation, the pain and discomfort associated with hemorrhoids, semi-solid rectal dosage forms are used, which provide better retention time in the rectum as compared to other dosage forms, and reduce patient compliance issues and increase the drug release [7].

### 2.3. Physiology Associated Factors

Physiological factors such as rectal mucus and the motility of the rectal wall will also affect drug absorption. Since the body is upright, the abdominal organs press onto the rectum which stimulates the spreading and promotes drug absorption.

#### 2.3.1. Rectal Fluid Volume and pH

In comparison to the small intestine, the rectal fluid volume is quite small (3 mL in normal conditions), which can interfere with drug dissolution and absorption. This limits the rate at which slightly soluble drugs are absorbed. Rectal pH is relatively neutral and aids the absorption of drugs with pKa values near or above the physiological range. The shift in the pH of the rectal chamber alters the degree of drug ionization and also causes irritation of the rectal mucosa, impacting drug absorption. The pH of the rectum can change with the administration of exogenous products due to the low buffering capacity of the rectal fluid [8].

#### 2.3.2. Presence of Rectal Contents

The presence of stool in the rectum can affect dissolution, stability, and drug contact with the mucosal wall for absorption, followed by irregular drug absorption [6]. The presence of fecal material inside the rectum is also one of the absorption limiting steps as stool presence will affect dissolution, stability, and drug contact with the mucosal wall for drug absorption followed by irregular drug absorption. The drug absorption will be higher when the rectum is empty.

#### 2.3.3. Rectal Mucous

Rectal mucus made of mucin and water forms a fluid layer that can act as a barrier for drug absorption. Drugs need to permeate across the mucus layer to reach the epithelial lining of the rectum. The retention time of the drug with the mucus layer also influences drug absorption. Since the body is upright, the abdominal organs press onto the rectum which stimulates the spreading and promotes drug absorption [6,8].

#### 2.3.4. Motility

The motility of the colon and the frequency of bowel moments is another factor that influences the absorption of drugs through the rectal route. The time of dosing must be considered with respect to a person’s bowel movements. Increased motility in conditions like diarrhea reduces the retention time of rectal dosage form which leads to lesser drug release and absorption.

### 2.4. Pathology Associated Factors

Pathological conditions like inflammatory bowel disease (IBD), hemorrhoids, gastro-intestinal infections, etc. can influence the efficacy of rectal drug delivery systems. This occurs due to variations in the integrity of tissues, inflammation of mucosa, and bowel motility. Diseases altering motility influence retention time, time available for disintegration, and absorption.

#### 2.4.1. Inflammatory Bowel Disease (IBD)

IBD causes mucosal inflammation, ulcers, and crypt distortions. This may reduce drug bioavailability and absorption due to accelerated colonic transit which gives lesser time for disintegration and dissolution.

#### 2.4.2. Haemorrhoids

Haemorrhoids are swollen veins occurring in the anal region. Local trauma and ruptured haemorrhoids can affect the integrity of rectal mucosa and may lead to enhanced drug absorption which can be painful to administer.

#### 2.4.3. Gastro-Intestinal Infections

Gastro-intestinal infections can be caused by various agents like bacteria, viruses, parasites, etc., and lead to diarrhea, i.e., an increase in intestinal motility, abdominal cramping, and so on. These infections lead to proctitis (inflammation) and can alter drug absorption from the rectum [9,10].

## 3. Rectal Diseases

Diseases of the rectum and anus are much more prevalent in the general population than those seen in clinical practice since most patients referable to anorectum disorders do not seek medical attention. Various kinds of drugs are employed for treating rectal disorders such as steroids (Hydrocortisone, budesonide, prednisolone), anti-inflammatory drugs (sulfasalazine, olsalazine, mesalazine, Balsalazine), anti-cancer drugs (5-Fluorouracil, bevacizumab, cetuximab, oxaliplatin), NSAIDs (Aspirin, sulindac, celecoxib) and hormones such as insulin and thyroid are also employed for peptide or vaccine delivery [11]. Rectal diseases are treated using various dosage forms as they are inexpensive to manufacture, self-administered by the patient, and offer improved drug availability (locally and systematically); the release kinetics (controlled or rapid release), drug targeting, and retention time are the reasons for rectal dosage forms requirement [12]. The rectal diseases are discussed below.

### 3.1. Perianal Abscess

Perianal abscess is a commonly occurring anorectal disorder where there is a collection of pus in the cavity near the anus and rectum. Most perianal abscesses occur due to the infection of the crypto globular glands which results in the formation of a cavity accumulating pus inside. The pus contains a mixture of dead tissue, immune cells, and bacteria (foreign particles) [13]. The pathogens/bacteria that cause perianal abscesses are aerobic and anaerobic microorganisms. If the bacterial infection goes through the external sphincter, it is called an ischiorectal abscess, and if it spreads to both sides of the rectum, it forms a horseshoe shape around the external sphincters. Symptoms may include anal pain, constipation, discharge, fever, and swelling [14].

### 3.2. Hemorrhoids

Hemorrhoids is a common disease related to the anal canal and rectum. Some vascular structures (normal blood vessels inside the rectum called internal hemorrhoids) are already present in the rectum. When these internal hemorrhoids swell, outgrow and start to bleed in the anal canal and outside the anus, they are termed “Hemorrhoids”, also known as Piles [15]. The causes of hemorrhoids are unclear, but constipation, lifting heavy weights, spicy food, irregular daily life schedule, pregnancy, and sitting on a toilet for a long time may be a few of the causes [16,17].

### 3.3. Anal Cancer

Anal cancer is a rare and malignant disease that affects the anogenital tract [18,19]. Tumors in the anal canal can be either keratinizing or non-keratinizing, depending on their position in regard to the dentate line [20]. It begins with the superficial mass, spreads locally, and may involve regional lymph nodes that show malignancy at distant organs. Various habits that cause anal cancer are cigarette smoking, receptive anal intercourse, genital warts, number of sexual partners during the whole life, and infection with Human Papilloma Virus (HPV) [21]. In most cases, the causative agent of anal cancer is the HPV infection [22]. In fact, 40% of anal cancer cases have a significant risk of HPV infection. Anal cancer mainly spreads via the lymph system and less commonly spreads through blood [23].

### 3.4. Fissure In-Ano

An anal fissure is a lining of cracks in the vertical of the squamous epithelium of the anal canal. An anal fissure rests over the inner sphincter of the anal canal. Chronicity of the anal fissure can be increased due to spasms of the sphincter present internally in the anal region. It is most painful due to the stretching of the upper layer anal region [24]. Its treatment involves the use of nitric oxide donors [25]. The conservative therapies of nitroglycerine, botulinum toxin, and nifedipine are conclusively beneficial approaches for managing chronic anal fissures, which can minimize the need for anesthesia and surgery in many patients [26].

### 3.5. Fistula In-Ano

Fistula-in-ano can be defined as the infected region between the perianal skin and the anus [27]. These are caused by an infection in the anal gland that spreads to the skin. The symptoms of fistula-in-ano include pain, swelling, and pus discharge from the anus [28]. The leading causes of fistula-in-ano are clogging of the anal gland, anal abscesses, Chron’s disease, radiation, STDs, tuberculosis, and cancer [29].

### 3.6. Anal Abscess

It is the most common anorectal disease seen in patients having inflammatory conditions like Crohn’s disease. It involves pus formation in the cavity of the anal region [30]. The origin of the abscess is a crypto-globular infection of the proctodeal gland present in the inter-sphincteric space [31]. The pain associated with anal abscesses is related to other concerns like swelling and redness in that particular area. The abscess finds the path of least resistance so, an abscess is formed at the gland terminal point [30]. It can be cured by simple drainage of pus alone, but in some patients, fistulotomy is required which implies anesthesia, surgery, and anal incontinence [32].

### 3.7. Anal Warts

Anal Warts are regarded as a viral origin disease, caused by HPV (Human Papilloma Virus). These are small tissue masses found inside and around the anus [33]. Initially, they appear as tiny spots or growths, but later grow and form a big bulge of tissue that covers the anal region [34]. These are caused by DNA containing the HPV virus family and can cause mucus discharge, bleeding, or itching from the tissue mass in some patients. Any direct contact with the infected anal part (fluid of the infected person) can cause anal or genital warts [33].

## 4. Rectal Drug Delivery Systems

**Figure 3:**
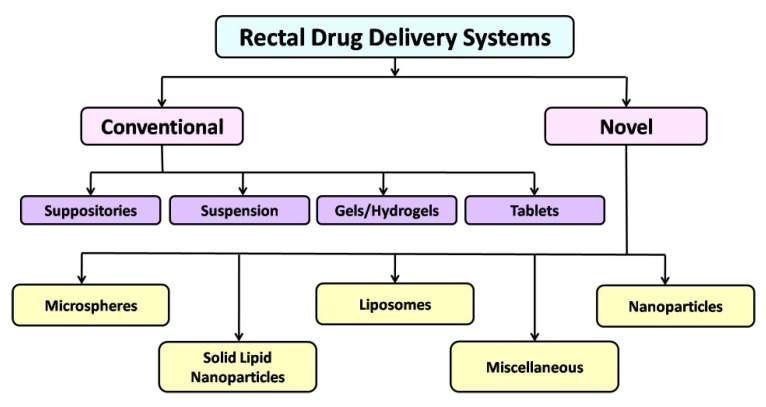
Classification of rectal drug delivery systems.

Various rectal drug delivery systems (RDDS) are available for treating rectal disorders. RDDS can be classified into conventional and novel delivery systems, as shown in Figure 3. The conventional delivery system includes suppositories, suspensions, gels, and tablets, whereas the novel drug delivery system includes polymeric micelles, nanoparticles, microspheres, and liposomes. The various delivery systems are discussed below.

### 4.1. Conventional Rectal Drug Delivery Systems

Due to acceptance and patient compliance issues, the rectal drug delivery system has not been investigated as much as other routes of drug administration such as oral, pulmonary, topical, etc. Rectal dosage forms have been developed for systemic and local action, and have been investigated for immediate or prolonged drug release. Nonetheless, rectal dosage forms are already available in the market and are available in liquids, semi-solids, and solid forms. This particular section will discuss the main conventional rectal dosage form and recent advances to improve their effectiveness [35].

#### 4.1.1. Suppositories

Suppositories are unit dosage forms injected into the rectum for systemic or local effects. Rectal administration can cause discomfort to patients, but it has benefits over other dosage forms used for gynecological and proctological diseases [36]. As per USP35 “A suppository is a type of solid dosage form having varying weights and forms of shapes mainly used for urethral ostium, rectal, or vaginal delivery, which usually melts, softens, and dissolves at body temperature”. Initially, it serves as a preventive or palliative agent or as a transporter of medicinal agents for systemic and local intervention. Rectal suppositories show a wide range of advantages and applicability but still possess certain drawbacks and challenges. Rectal suppositories may cause irritation and can be uncomfortable for the patients. Other challenges are issues pertaining to storage and packaging, as some suppositories may need a refrigerator to store them [37].

The suppository base determines the pharmacokinetic and pharmacodynamic characteristics of the suppository. USP classifies suppository bases into six different categories namely, cocoa butter, cocoa butter substitute, polyethylene glycol, glycero-gelatin, surfactant, tablet suppositories, or inserts. Suppository bases can also be classified based on the melting property or their dissolution. Suppository bases such as fat or oil melt at body temperature, while glycerol-gelatin bases absorb water and dissolve to release API. A suppository is a set of bases that includes a dissolving agent, natural resins, fast-dissolving agents, collagen, fibrin, hydrogels, and other water-soluble or water-miscible polymers or surfactants. The physicochemical characteristics of the APIs used for suppositories have an impact on the base utilized in the suppository formulation [38].

In a study, an artemether self-micro emulsifying suppository (SMES) was prepared for faster onset of action and prolonged effect. SMES showed increased antimicrobial activity against the malaria parasite *Plasmodium berghei* up to 94% for 20 days post-infection. Additionally, animal survival was found to be higher in comparison to the traditional formulation [39]. Another study showed the preparation of gelling carbamazepine liquid suppository that was thermally reversible to avoid hepatic first-pass clearance. According to the release mechanism study, CBZ was released via fickian diffusion from the suppositories. In comparison with the oral solution containing the same quantity of drug, the in vivo study revealed a greater peak plasma concentration of CBZ via suppository and suggested an effective drug delivery system [40]. The pharmacokinetic parameters of diclofenac sodium suppositories were compared with oral enteric-coated and SR tablets. The absorption time of suppositories was about 4.5hrs. However, the defecation process may remove the drug, leading to a suppository’s low relative bioavailability (55%). At the same time, sustained-release formulation shows slow first-order absorption and obeys the flip-flop model since the disposition rate constant is more than the absorption rate constant [41].

Kauss et al., formulated an azithromycin suppository for paediatric use. According to the in vivo study on a rabbit, azithromycin was delivered as a solid solution suppository and showed a bioavailability of 47%, which was higher than oral product in humans (38%). The stability and feasibility studies were compatible with industrial production scale-up [42]. A researcher formulated a nanostructured lipid carrier-based ondansetron suppository for its enhanced rectal absorption and in vitro and in vivo evaluations were performed. Suppositories enhanced drug absorption and offered prolonged drug release [43]. In one of the studies, mesalazine suppositories were formulated for active ulcerative proctitis and compared with oral mesalazine. These research findings showed that in the therapy of acute ulcerative proctitis, mesalazine suppositories showed better outcomes than oral mesalazine [44]. Researchers also developed an in-situ-gelling and injectable Pluronic–poly (acrylic acid) (Pluronic–PAA) liquid suppository. When oxaliplatin was added, the toxic effects were studied, and cytotoxic tests showed that Pluronic and PAA were non-toxic substances that could suppress colon cancer cells. These results suggest Pluronic–PAA liquid suppository can minimize the toxicity of anti-cancer medications by avoiding the first-pass metabolism, in-situ-gelling, and injectable liquid suppository for people as a more convenient and effective rectal administration method [45].

Nowadays, hollow-type suppositories and thermos-responsive liquid suppositories are also being developed. Hollow-type suppositories were discovered in the 1980s, which contain a hollow cavity that can accommodate either solid, liquid, or gel inside. These can accommodate thermolabile drugs and rapid drug release can be achieved as the drug can be incorporated into both either a shell or hollow cavity. Piroxicam and bisacodyl are used for developing hollow-type suppositories [46,47]. In comparison to conventional suppositories, hollow-type suppositories showed rapid drug release. Thermo-responsive liquid suppositories are another advancement in suppositories, with thermos-responsive rectal gels that convert to liquid at physiological temperature to release the drug slowly for localized or systemic action. These are easy to administer and offer sustained drug release. Tolmetin sodium (NSAIDs) marked side effects on the gastro-intestinal tract on oral administration. Hence, thermo-responsive liquid suppositories of tolmetin sodium were prepared, it showed no morphological damage to the rectum, and a 4-fold increase in bioavailability was also observed [48].

#### 4.1.2. Rectal Suspension

A rectal suspension (also known as an enema) is a heterogeneous fluid mixture containing enough solid particles to cause sedimentation. The particles can be greater than one micrometer and would settle gradually, but the mixture is only known as a suspension if the particles have not settled out. These are applied to the rectal area to have a local or systemic effect and for diagnostic purposes. They contain excipients to adjust the viscosity, pH, increase the solubility of the active ingredient(s), and stabilize the preparation. These are packaged in single-dose tubes with volumes of 2.5 to 2000 mL. The bottle is either designed to administer the preparation to the rectum or comes with an applicator [49]. Rectal suspensions can hurt when the bottle tip is inserted, and the rectal temperature can influence the absorption of the drug.

Investigation of suppositories and rectal suspensions for their medicinal function and clinical pharmacology found that acetaminophen (20 mL) suspensions are more readily absorbed than suppositories. The bioavailability of 1 g acetaminophen in a 20 mL suspension in the rectal cavity is 90–91% compared to the oral dosage form (relative bioavailability). Another study for rectal indomethacin solutions showed higher relative bioavailability than oral indomethacin (112–137%). The rectal solution of ibuprofen was found to have relative bioavailability of 88% as compared to oral dosing, with a T_max_ of 1.1 h which was 0.33 h for oral dosing [50]. From 2017 to 2020, the treatment outcomes of rectal suspension of topiramate in three patients aged one year were studied, and observed no side effects or rise in seizure frequency [51]. Donnelly R. F. prepared rectal suspensions of levodopa and carbidopa and examined them for their stability. The results revealed that solutions were simple to resuspend, no caking had occurred, and the pH did not change for 35 days of storage at either temperature [52]. In one of the case studies, the impact of carbon nanoparticle suspension injection was investigated on rectal cancer patients before 30 min of operation. The investigation showed a reduced extent of lateral lymph node dissection in some patients and improved pathologic staging [53]. Another case study of the intra-rectal use of epinephrine suspension in prostate cancer radiotherapy was investigated. As per the studies, no variations in systolic blood pressure and heart rate were observed at any time point. No rectal toxicity after a 2-year follow-up was observed. Hence the studies concluded that intra-rectal epinephrine administration in prostatic radiotherapy is feasible and effective [54].

#### 4.1.3. Tablets

Tablets are a composition of appropriate excipients to form solid unit dosage forms. It is composed of a powdered mixture of active substances and excipients compressed or compacted into a solid dosage. Rectal tablets are single-dose medications dissolved or dispersed in water or other appropriate solvents before being administered to form rectal solutions or suspensions.

In research, the pharmacokinetic profiles of misoprostol tablets given rectally, orally, and vaginally in pregnant women were compared. Vaginal misoprostol stayed in the bloodstream longer than oral misoprostol, with a greater area under the curve at 240 min. At 240 min, rectal misoprostol had a similar pattern but a much lower area under the curve. They also found that using 800 g of rectal misoprostol regularly reduced blood loss following delivery. The regimen was recommended for low-resource, high-volume obstetric conditions [55]. Shojai et al. carried out research on five patients for delivery-induced hemorrhage by rectal misoprostol tablet administration. The hemorrhages ended in less than 5 min and had no immediate side effects [55]. In one of the research projects, the pharmacokinetic properties of Lamotrigine tablets after oral and rectal administration in human volunteers were compared. The relative bioavailability of the drug was found to be 0.63–0.33 after rectal administration with no severe side effects [56].

#### 4.1.4. Gels and Hydrogels

As compared to liquid formulations, semi-solid dosage forms are better maintained in the rectal cavity. Semi-solid formulations show faster drug release than solid suppositories, and no lag time is required for dissolution or melting, and immediate pharmacological action. Drug release with semi-solid dosage forms is widely used for localisedtreatment such as lower bowel inflammation and haemorrhoids [57]. The most widely used semi-solid dose forms for rectal drug delivery are gels, hydrogels, and ointments. The gel shows better spreadability and stability properties than ointments and creams. Gels and ointments are typical rectal dosage forms. The drugs are dispersed equally in hydrophilic or lipophilic bases and excipients such as Tween^®^ 80 and glycerine to increase absorption. Viscosity can be enhanced by incorporating co-solvents (e.g., propylene glycol and glycerine) or electrolytes [58].

Gels (sometimes called jellies) are a semi-solid system in which a liquid phase is constrained within a three-dimensional polymeric matrix having a high degree of cross-linking [59]. Gels are jelly-like semi-solid structures made by the dispersion of tiny or big molecules in an aqueous liquid medium by adding a gelling agent. Gelling agents used are synthetic macromolecules such as carbomer 934, which are of high molecular weight. Gelling agents used are cellulose derivatives, such as hydroxypropyl methylcellulose or carboxymethyl cellulose, and natural gums, such as Tragacanth [60]. Rectal gels need to be packed with special perforated plastic tips. The use of gels helps in longer retention of the drug.

A cross-linked 3D (three-dimensional) assembly that absorbs a substantial amount of aqueous solution causing swelling of the network is known as hydrogel. The unique physical preparations have gained great interest in their uses in drug delivery. The unique characteristics of hydrogels for their use in drug delivery include controllable swelling behaviour, high water content, ability to control drug release, ease of handling, and biodegradability. It also provides a design that offers favourable conditions for the therapeutic area to achieve a medicinal impact and avoid side effects [61]. Natural gums gelatin, polyacrylates, cellulose derivatives, and some other polymers can be applied to the formation of hydrogel systems. Hydrogels are like living tissues because they retain more aqueous solutions, swelling properties, and smooth consistency. The hydrogel is more elastic and more potent than available hydrogels of similar softness. Poly (methyl acrylate-co-hydroxyethyl acrylate) hydrogel implant material of strength and softness [62]. Ciolacu et al. developed insulin-loaded binary hydrogels of methylcellulose and polyacrylate to prevent type I diabetes in the form of a rectal suppository. Animal experiments found that the hypoglycemic activity of the INS-loaded hydrogel was evident. This technique of administration could improve diabetic patients’ compliance. Finally, it could be speculated that binary hydrogel was used to treat type I diabetes through rectal administration [63].

A mucoadhesive hydrogel of sulfasalazine (SSZ) made up of genipin-crosslinked catechol modified-chitosan (Cat-CS) was prepared to enhance SSZ efficacy via the rectal route. As compared to oral SSZ, rectal SSZ showed equivalent histological scores, improved therapeutic efficacy, and lower toxicity in the ulcerative colitis mouse model. Conclusively, rectal SSZ hydrogels were found to be more effective for the treatment of ulcerative colitis [64]. In a study 5-aminopyrazole conjugated gelatin hydrogel was prepared to load 5-fluorouracil. The hydrogels showed predictable drug release patterns in simulating rectal conditions along with notable cytotoxicity against human colon adenocarcinoma HT29 cells [65].

In the case of acute seizures, benzodiazepines are the recommended therapy. Hence, one of the researchers formulated rectal hydrogels containing diazepam and was evaluated. The prepared gel showed good drug content (96–103%), excellent anti-microbial activity, and viscosity [66].

Researchers developed mucoadhesive and thermosensitive rectal gels of quinine for paediatric patients and evaluated them in rabbits. The bioavailability of mucoadhesive gels was found to be greater than thermosensitive hydrogels. Additionally, the in vivo studies showed sustained release from both the gels, and no damage to rectal mucosa of the rabbit was observed [67]. In another study, indomethacin gels were prepared using pluronic F-127 and administered to the rabbit via the rectal route. The gel did not produce a sudden peak in plasma concentration but instead, a sustained effect was observed from 10 to 15 h. As a result, indomethacin formulation based on PF-127 aqueous gels appeared to be an effective rectal preparation with long-acting action and fewer side effects [68]. Diastat^®^, a marketed rectal gel containing diazepam, has been successfully developed and was found to deliver diazepam to the systemic circulation efficiently. This formulation was effective in treating acute repeated seizures (ARS) since it improves the duration between convulsions by 12 h. Diastat^®^, administered as a single rectal dose, was found more effective than a placebo in reducing the number of seizures that occurred during an ARS episode [69].

### 4.2. Novel Rectal Drug Delivery Systems

The novel rectal drug delivery systems are investigated to improve the therapeutic efficacy of a drug for both local and systemic action of the drug. The conventional RDDS differs from novel RDDS in terms of formulation properties (such as spreadability), release characteristics, retention, and pharmacokinetic profile. Novel RDDS includes encapsulation of the drug into the carrier system before dispersion into any other base; this allows improved solubility and protects the drug from degradation. They also offer better control over spreadability, prolonged retention, and controlled drug release via different dosage forms [70].

**Figure 4:**
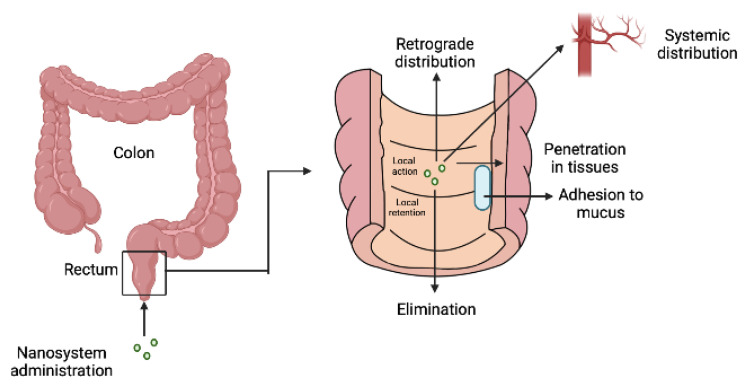
Mechanism of drug release of the nanosystems in rectum.

A nanotechnology-based drug delivery system offers significant advantages for the rectal administration of active compounds. Nanosizing is helpful in enhancing the therapeutic efficacy of insoluble drugs. Rachmawati et al., demonstrated enhanced anti-inflammatory effects of curcumin nanosuspension stabilized with D-α tocophennol polyethylene glycol after rectal administration in a colitis rat model [71]. Nanocarriers are further helpful in developing physicochemically stable systems for rectal administration of heat, liable compounds such as proteins/nucleic acids. Depending upon the type, nanocarrier systems could be developed for enhancing the retention, transport, and distribution of drugs across the rectal mucosal surface for increasing therapeutic efficacy. Moqejwa et al. developed nanosized trasferosomes for enhanced delivery of cannabidol after rectal administration. The researcher prepared tizanidine-loaded nanotransferosomes for rectal administration with the aim of bypassing the hepatic first-pass metabolism. The nanotransferosomes demonstrated prolonged drug release with enhanced bioavailability [72]. Another advantage of a nano-based delivery system is drug targeting. Targeted drug delivery significantly improves the efficacy of the drug with a potential reduction in side effects and drug release. Targeted drug release is generally a three-step process: (i) binding of the nanocarriers with the receptors of the target cell, (ii) endocytosis-based entry of the nanocarriers system with the cell, and (iii) drug release. Seo et al. developed docetaxel-loaded thermosensitive and bioadhesive nano-micelles for improved bioavailability and chemotherapeutic effect [73]. Biologically, novel RDDS ensures better cellular uptake into mucosal cells and tissues, promotes drug accumulation at target sites, more uniform distribution, and drug release within the rectal region. The drug release from the nanosystem follows transcellular or paracellular pathways for permeability across the epithelium. For drug absorption, it should first dissolve (in mucin) and diffuse across the mucosa. Mucin can be one of the natural barriers in drug absorption of poorly soluble molecules or DDS. On reaching the mucosa, the drug may retain for local action or further penetrate the mucosa, crossing the epithelial lining and reaching the blood vessels for systemic circulation [74], as shown in Figure 4.

#### 4.2.1. Rectal Microspheres

Presently, mucoadhesion is a hot topic for the development of drug delivery systems. Microspheres are small spherical bodies with a particle size range of 1–1000 µm and are composed of biodegradable and non-biodegradable materials. These have a long residence time allowing a direct relationship with the underlying absorption surface and improving therapeutic drug performance [75]. It allows precise delivery of potent drugs and lower drug concentrations at locations other than the target site, and ensures the safety of labile compounds before, during, and after administration and before their presence at the site of action. Microsphere manufacturing is a challenging process due to the properties of the polymers encapsulating the drug to be administered. Other challenges faced are microsphere filtration, reproducibility, and consistency [76].

A group of researchers came up with a novel way of formulating mucin-gelatin mucoadhesive microspheres for rectal ceftriaxone sodium delivery and evaluated the microspheres. The results indicated that ceftriaxone sodium can be inserted in microspheres made of both type A gelatin alone and its admixtures with porcine mucin and were delivered rectally [77]. In one of the studies, the basic emulsification cross-linking technique was used to prepare mucoadhesive microspheres rectal suppository. The drug content of suppositories was found to be between 70.94 and 91.65% and at the end of 10 h, suppositories were found to delay drug release [78]. A unique mucoadhesive hydrogel loaded with diclofenac sodium–chitosan microspheres were formulated for rectal administration. The physicochemical studies showed that the hydrogels have a pH of 6.5–7.4 and were ideal for rectal use. The in vitro drug release was found to be 34.6–39.7% after 6 h and showed negligible irritant reaction histopathologically [79].

Mesalazine-containing chitosan microparticles were formulated for rectal administration to improve inflammatory bowel disease (IBD) clinical therapy. To increase the drug’s anti-inflammatory efficacy, mesalazine was entrapped within the particles of chitosan by employing the polysaccharide’s bio-adhesive feature. In vitro and in vivo tests confirmed the therapeutic efficiency at a 2-fold lower drug dose than the generic formulation Asamax^®^ [80]. Kietzmann et al. developed pH-sensitive microspheres of carboxyfluorescein (CF) for rectal delivery in male Wistar rats induced with colitis. The oral bioavailability of CF solution was reduced by colitis as compared to stabilised controls, and equivalent findings were observed when CF solution was administered rectally. However, CF-microspheres led to a higher local drug concentration in the colonic tissue [81].

#### 4.2.2. Nanoparticles

Spieser and colleagues created nanoparticles as a drug delivery vehicle in the late 1960s. In the early 1970s, nanoparticles of cross-linked polyacrylamide were produced. A study emphasised the convergence of radiochemistry for imaging and therapy with advances in nanoparticle (NP) design for biomedical applications [82]. Furthermore, the magnetic particles were incorporated within nanoparticles, using a magnetic field for targeted drug delivery. Nanoparticles are natural or artificial polymers ranging from 50–500 nm in size [83]. These comprise macromolecular materials in which the active moiety (drug or biologically active material) is entrapped, dissolved, and or to which the active principle is attached or adsorbed. Mainly there are two types of nanoparticles namely, nanospheres, and nanocapsules. Nanospheres are solid core spherical particulates containing drugs embedded within the matrix or adsorbed onto the surface (Matrix type) whereas, nanocapsules are vesicular systems encapsulating the drug in the central core surrounded by a polymer sheath (Reservoir type). Nanoparticles possess a challenge in that they are one of the most complicated delivery systems. They can be prone to surface and bulk erosion and there may be a loss of initial particle characteristics [84].

In a research study, both in vivo and ex vivo evaluations were performed to study polymeric nanoparticles of the anti-HIV drug dapivirine for vaginal and rectal delivery using poly (ethylene oxide) (PEO) as a polymer. Increased drug retention was observed in all nanoparticles as compared to pure dapivirine. The in vitro toxicity was also reduced by PEO modification [85]. A study carried out by Maisel et al., demonstrated the effect of mucoadhesive nanoparticle and non-mucoadhesive nanoparticle (muco-penetrating nanoparticle) interaction with gastrointestinal mucus and distribution in the gastrointestinal tract by oral and rectal administration in the mouse. The non-mucoadhesive nanoparticles showed loose contact with the epithelium and penetrated much more efficiently into the inflamed region of ulcerative colitis. However, the nanoparticles administered via the rectal route showed increased drug distribution than the oral route [86].

The rectal route is also suitable for the delivery of anti-viral drugs for treating viral diseases such as HIV. Nunes et al. developed PLGA (polylactic-co-glycolic acid) nanoparticles of efavirenz and tested them for mucus-diffusive behaviour on rectal administration in mice. The nanoparticles retained antiretroviral activity with low toxicity against epithelial cells and HIV target cells. Additionally, the nanoparticles showed increased bioavailability of the drug compared to pure efavirenz [87]. In order to treat ulcerative colitis, the meselamine nanoparticles were prepared and proved their potential to retain the drug from systemic absorption and also reduced inflammation [88]. In one more study curcumin nanoparticles with a size of approximately 200 nm were successfully produced and a seven-fold increase in bioavailability was observed. Improved anti-inflammatory effect in ulcerative colitis was obtained on low doses [71].

#### 4.2.3. Liposomes

Liposomes are a novel drug delivery system that resembles vesicular structures made up of bilayers that spontaneously form when phospholipids are strewn around in the water. They’re tiny vesicles with a membrane made up of lipid bilayers that enclose a fluid amount. Liposomes have been used to enhance the therapeutic index of experimental and current medications by modifying medication retention, increasing metabolism, extending cellular half-life, and reducing toxicity. The polar design of the liposomal centre allows the typification of polar drug molecules. According to their tolerance for phospholipids, lipophilic, and amphiphilic molecules are solubilised within the phospholipid bilayer. Niosomes are formed when non-ionic surfactants are used instead of phospholipids in a bilayer structure. Standard phospholipid bilayer membrane-based “first-generation liposomes” had clearance and low stability after injection. This is because physical encounters with protein adsorption and circulating proteins in the blood lead to their clearance, significantly affecting traditional liposome membranes. Longer-circulating liposomes were developed by changing the structure, scale, and charge of regular liposomes to resolve these deficiencies [89,90].

In a study, ferritin (a soluble model antigen) was used to test whether liposomes can provide an effective delivery vehicle for mucosal immunization via the rectum. The findings suggested that liposomes and immune adjuvants may be used to immunize mucosa through the rectum, cholera toxin is an immunoadjuvant that works well in the rectal colonic mucosa, IgA appeared to increase liposome uptake by M cells, enhancing the local secretory immune response to antigen in liposomes [91]. In another study, the identification of liposomes in the brain, liver, and spleen after rectal administration was examined. The position of the liposomes in the brain following rectal administration demonstrates that this procedure can effectively cross the brain-blood barrier. Furthermore, the chance of embolism and hypersensitivity, as well as tight sterility regulation and a variety of other adverse effects, can be avoided [92]. Rectal administration can play a vital role in producing liposome drug-entrapped treatment and diagnostics. In a study, 5-fluorouracil liposomes were prepared and evaluated in vitro on cell lines. Results showed an enhanced cytotoxic effect of 5-fluorouracil as compared to pure 5-fluorouracil and found to potential carriers for rectal administration [93].

#### 4.2.4. Solid Lipid Nanoparticles

Solid Lipid Nanoparticles (SLNs) are the other most important form of nanoparticles, which are tiny spherical articles made by melting solid lipid nanoparticles made by melting solid lipids in water with the addition of an emulsifier to form stabilized solution. The particle size of SLNs ranges from 50–1000 µm. Drugs with poor pharmacokinetic, poor physiochemical compatibility, and heat liable drugs can be delivered via SLNs. These are very useful in controlled, targeted, and sustained drug delivery. In a study SLN of diazepam were prepared and showed prolonged drug release [94].

Ibuprofen (IBU), a non-steroidal anti-inflammatory drug, has poor gastrointestinal absorption due to low aqueous solubility. To overcome this problem, thermosensitive in situ gel loaded with ibuprofen solid lipid nanoparticle were developed for rectal administration. In comparison to IBU-SLN, the IBU-SLN-ISG showed a biphasic release pattern with initial burst release followed by sustained release. The gel showed better absorption and improved bioavailability in rats with no irritation and damage to rectal tissues. The gel is also retained for a longer period of time [95]. Topotecan is a synthetic derivative of Camptothecin which is used in colorectal and small lung cancer. In a study SLNs of topotecan were developed and incorporated into a thermoresponsive hydrogel system. The gel was administered to the rat rectum and showed controlled drug release over an extended period of time. The pharmacokinetic studies showed increased bioavailability of drugs with improved plasma concentration and anti-tumor effect [96]. In one of the studies, SLNs of irinotecan were formulated and loaded into the double reverse thermoresponsive hydrogel. The SLNs showed an entrapment efficiency of about 93% and a particle size of about 180 nm. The SLN-loaded hydrogels were easily administered in the body, quickly gelled, and formed a strong gel [97]. Additionally, the solid lipid nanoparticle of flurbiprofen-loaded dual-reverse thermosensitive hydrogel (DRTH) was also prepared for rectal administration with improved bioavailability and reduced initial burst effect. The formulation was easily administered in the rat rectum and increased drug dissolution rate and plasma concentration was observed. Moreover, there was no damage to rectal mucosa with improved bioavailability and a reduced initial burst effect was identified [98]. Novel dual-reverse thermosensitive solid lipid nanoparticle-loaded hydrogel for rectal administration of flurbiprofen with improved bioavailability and reduced initial burst effect. Sznitowska et al. formulated solid lipid nanoparticles of diazepam for rectal administration in rabbits. The studies concluded that the relative bioavailability of SLN was low (47%) compared to the aqueous organic solution [99].

#### 4.2.5. Miscellaneous

In this section, work carried out on miscellaneous novel nanosystems like niosomes, nanotransferosmes, and nanomicelles for rectal drug delivery have been presented and discussed.

In one of the research pro-niosomal gel of rutin was developed to treat haemorrhoids locally. They concluded that surfactant concentration inhibits entrapment and drug release. The pro-niosomes showed maximum drug release (up to 98%) and maximum drug deposition in the rectal walls [100].

Nano-transferosomes were recently used to examine another vesicular drug delivery technique. Nano-transferosomes are liposomes that employ edge activators for obtaining ultra-flexible activity. The transferosomes possess much flexibility and deformability activity due to the edge activators. The best transferosome formulations were found of tizanidine blended with hydroxypropyl methylcellulose (HPMC) in a pluronic-based thermoreversible gel. The pharmacokinetics analysis revealed two times more bioavailability and a longer half-life than the oral drug in rabbits [101]. In a study, nano-sized transferosomes of cannabidiol were formulated for rectal drug delivery. The transferosomes showed stability at up to six months at room temperature with particle size ranges from 102.2–130.1 nm. Ex vivo permeation studies revealed that the transferosomes improved the diffusivity and permeation across the excised colorectal membrane [72].

Nanomicelles are self-assembling nanosized (usually with particle size within a range of 10 to 100 nm) colloidal dispersions with a hydrophobic core and hydrophilic shell. These are currently used as pharmaceutical carriers for solubilizing hydrophobic drugs. Researchers investigated thermosensitive and bioadhesive nanomicelles of docetaxel for rectal administration with an aim to enhance its bioavailability and chemotherapeutic effect. Results revealed a 29% increase in the bioavailability of nanomicelles as compared to pure docetaxel. The rectally administered nanomicelles exhibited better chemotherapeutic effects than pure drugs [73]. Different types of novel drug delivery systems for rectal administration along with the incorporated drug and key outcomes are depicted in Table 1.

## 5. Rectal Formulations in Clinical Trials

Despite of most convenient drug administration route (oral), there are some situations where drug administration via the oral route is not possible. In such cases, the rectal route is the most favorable route, as it helps to deliver drug doses for both systemic and local actions. The rectal route also bypasses the hepatic metabolism, improves drug bioavailability, and offers controlled and sustained drug release. Nowadays there are great advancements in the optimization of rectal formulation, even though a few of them are able to reach the clinical phase of drug trials. Several clinical trials are going on/performed for rectal formulations which are listed below, in Table 2, Table 3, Table 4 and Table 5.

The rectal route could be considered as an alternative to the oral route for drug administration to paediatric patients as swallowing and taste masking issues are overcome. Moreover, drug administration is favourable in the case of vomiting, unconscious, and emergency patients. The main disadvantage of rectal drug delivery is poor acceptability and patient compliance along with low absorption capacity and high inter-individual variabilities of drug bioavailability. Maeda et al. performed a clinical study comparing the pharmacokinetic performance of azithromycin through oral and rectal administration in paediatric population. An azithromycin suppository administered through the rectal route demonstrated significantly increased bioavailability when compared with oral administration [102]. In another study, randomized clinical trials compare buccal midazolam with rectal diazepam in the treatment of prolonged seizures in Ugandan children. Buccal midazolam was found to be safe and more effective than rectal diazepam for the treatment of seizures in Ugandan children [103].

To improve the acceptability, compliance, and therapeutic outcome of rectal dosage forms in paediatric population efforts are made for improving dosage form requirements, production, marketing, and education regarding the benefits and usage of rectal formulations. High-dose acetaminophen and diclofenac-based suppositories were prepared to compare their antipyretic activity in paediatric patients during a randomised clinical trial. The study concluded that both the rectal suppositories reduced the rectal temperature significantly, but diclofenac suppositories reduced temperature more effectively than high-dose acetaminophen suppositories [104].

The effective use of a drug delivery system may be compromised in geriatric patients due to variations in physiology, comorbidities, physical/ mental deterioration, and the use of multiple drugs. In a study of six geriatric hospital patients, the pharmacokinetics of diazepam given as suppositories or as a solution in rectal tubes was studied. From the study, it was concluded that there was no significant difference in bioavailability of these formulations, concentration, or duration to reach the maximum [105]. Hagen et al. studied the absorption of paracetamol from suppositories in geriatric patients with faecal accumulated in the rectum. The study concluded that the paracetamol concentration was significantly lower in the patients with faecal matter in the rectum [106].

Further on, some more clinical trials conducted in the field of rectal drug delivery are enlisted in Table 2, Table 3, Table 4 and Table 5 [107].

Various clinically approved rectal formulations for local and systemic action are tabulated in Table 6 [108].

## 6. Recent Advancements and Patented Formulations in Rectal DDS

The rectal route has been extensively explored for the delivery of pharmaceutical and herbal bioactives for the diagnosis and treatment of local and systemic conditions. Different types of nanocarriers including nanoparticles, micelles, liposomes, and nanoemulsions have been researched for delivery through the rectal route. Lipofectamine^®^ is a commercially available liposome-based formulation for the intranasal administration of siRNA [109]. 3D printing technology is an emerging field developing diverse drug delivery systems with favourable characteristics for the desired route of administration. The development of modified polymers is another key area where the researchers are working to develop sustainable, stable, economical polymers for developing rectal drug delivery systems with customized properties. Advancements and applications of insulin tools for the production of PKPD, and IVIVC are also helpful in the development of site-specific drug delivery systems.

The patented formulations of the rectal dosage form are shown in Table 7.

## 7. Conclusions

The rectal route offers a potential alternative for the delivery of pharmaceutical actives because of its potential advantages. Major drawbacks of rectal drug administration are low patient compliance, small absorption area, pathological conditions, interruption in drug absorption by defecation, and rectal irritation. Nano-based approaches and the use of novel polymers could offer potential therapeutic advantages for local and systemic drug delivery. Smart medical devices could also see the light of day in the prognosis, diagnosis, and treatment of rectal disorders. Novel drug delivery systems are offering significant advantages for rectal administration making this route, a favourable one for better patient compliance and therapeutic efficacy. Nanocarriers have been found to be suitably therapeutically effective for the management of local and systemic drug delivery through rectal administration. However, more understanding of the retention, absorption, and distribution of nano-systems through the rectal biological membrane is required for developing a sustainable drug delivery system. Moreover, the safety and toxicity of nano-systems and other novel drug delivery systems must be defined, illustrated, and addressed on a case-by-case basis. The development of modified polymers with desirable quality attributes is also favouring the development of innovative rectal drug delivery systems. If the compatibility, toxicity, and regulation issues of the rectal drug delivery system are adequately addressed, this route could become a preferred route for the treatment of various diseases/disorders.

## Figures and Tables

**Table 1:** Novel drug delivery systems for rectal administration.

S. No.	Nanosystem	Drug/Moiety	Key Outcomes	Ref.
1	Nanoparticle	Curcumin	A seven- fold increase in bioavailability was observed.	[71]
2	Nano-transferosomes	Cannabidiol	Nano-transferosomes showed improved diffusivity and permeation across excised colorectal membrane.	[72]
3	Thermosensitive and bioadhesive nano-micelles	Docetaxel	Nanomicelles showed the ability to improve bioavailability and chemotherapeutic potential of Docetaxel in vivo.	[73]
4	Mucoadhesive microspheres	Ceftriaxone sodium	The in vivo studies in male Wistar rats revealed increase drug release and bioavailability of drug.	[77]
5	Mucoadhesive microspheres rectal suppository	Alverine Citrate	Sustained drug release was observed and found useful in treating inflammatory bowel syndrome.	[78]
6	Mucoadhesive hydrogel microsphere	Diclofenac sodium	34–39% drug release was observed at the end of 6 h and no irritation was observed histopathologically.	[79]
7	Microparticles	Mesalazine	Microparticles showed efficient drug retention and the in vitro and in vivo studies confirm its mucoadhesion and therapeutic efficacy at a lower dose (13 mg/kg) than marketed formulation (26 mg/kg).	[80]
8	pH-sensitive microspheres	Carboxyfluorescein	The microspheres led to a higher local drug concentration in the colonic tissue.	[81]
9	Polymeric nanoparticles	Dapivirine	Increased drug retention was observed on rectal administration than pure drug.	[85]
10	Mucoadhesive nanoparticle and non-mucoadhesive nanoparticle	-	Nanoparticles administered via rectal route showed increased drug distribution than oral route.	[86]
11	PEG CoatedNanoparticle	Efavirenz	The efavirenz nanoparticle were found to be safe after once daily administration for 14 days.	[87]
12	Nanoparticle	Meselamine	The nanoparticles absorbed and retain for much longer time providing systemic drug action. The inflammation produced by UC was also reduced.	[88]
13	IgA coated Liposomes	-	The coloniclrectal IgA response to liposomal ferritin was significantly enhanced.	[91]
14	Bangham-type liposomes	-	Rectal administration of liposome showed that blood-brain barrier can be overcome.	[92]
15	Liposomes	5-fluorouracil	Enhanced cytotoxic effect of 5-fluorouracil as compared to pure 5-fluorouracil and found to potential carriers for rectal administration.	[93]
16	In situ gel loaded with solid lipid nanoparticle (ISG-SLN)	Ibuprofen	In comparison with IBU-SLN, IBU-SLN-ISG showed initial burst release followed by sustained release and produced much better absorption of IBU and improved bioavailability in rat with no irritation or damage to rectal tissues, and retained in the rectum for a long time.	[95]
17	SLN-Loaded Thermoresponsive Hydrogel	Topotecan	In vivo studies in rat rectum showed controlled drug release over extended period of time. The SLNs showed improved bioavailability, plasma concentration, and anti-tumor effect with no toxicity.	[96]
18	Irinotecan-solid lipid nanoparticles loaded double reverse thermosensitive hydrogel (DRTH)	Irinotecan	The DRTH showed easy administration, fast gelling, and strong gel-forming in the body.	[97]
29	Dual-reverse thermosensitive solid lipid nanoparticle-loaded hydrogel	Flurbiprofen	Increased drug dissolution rate and plasma concentration were observed. No damage to rectal mucosa with improved bioavailability and reduced initial burst effect was identified.	[98]
20	Solid lipid nanoparticle	Diazepam	The relative bioavailability of SLN was low (47%) compared to the aqueous organic solution.	[99]
21	Pro-Niosomes	Rutin	The pro-niosomes showed maximum drug release (up to 98%) and maximum drug deposition in the rectal walls.	[100]
22	Transferosomes	Tizanidine	2 times more bioavailability and a longer half-life via rectal route than the oral administered drug in rabbits.	[101]

**Table 2:** Clinical trial outcomes of suppositories-based drug delivery for rectal administration.

S. No.	Drug	NCT Number	Condition	Status
1	NRC001	NCT00857467	Fecal Incontinence	Completed
2	NRC001	NCT01265485	Fecal Incontinence	Completed
3	NRC001	NCT00893607	Fecal Incontinence	Completed
4	NRC001	NCT01175941	Fecal Incontinence	Completed
5	Nifedipine	NCT00972907	Chronic Anal Fissure	Completed
6	Nifedipine	NCT02023047	Chronic Anal Fissure	Completed
7	Budesonide	NCT01966783	Proctitis	Completed
8	Mesalamine	NCT01172444	Proctitis	Terminated
9	AnucortHC	NCT01913158	Internal Hemorrhoids	Completed
10	Hydrocortisone Acetate	NCT03335774	Internal Hemorrhoids	Completed
11	Asacol	NCT05091775	Fissure in Ano Diarrhea	Completed
12	Hydrocortisone acetate	NCT04469686	Ulcerative Proctitis	Recruiting
13	Flucortolone & Lidocaine	NCT03757078	Acute Hemorrhoids	Completed
14	MAX-002	NCT01016262	Proctitis, Ulcerative	Terminated
15	Dendrobium Huoshanense	NCT05079438	Locally Advanced Rectal Cancer	Recruiting
16	1R, 2Smethoxamine hydrochloride	NCT01656720	Faecal Incontinence	Completed
17	Bisacodyl	NCT02609607	Constipation Fecal Incontinence Multiple Sclerosis	Terminated

**Table 3:** Clinical trial outcomes of microspheres, nanoparticle, liposomes-based drug delivery for rectal administration.

S. No.	Drug	NCT Number	Condition	Status
**Microspheres**				
1	Irinotecan	NCT03086096	Colorectal Carcinoma Neoplasm Metastasis	Completed
2	FOLFOX	NCT00724503	Colorectal Cancer Colorectal Carcinoma Liver Metastases	Completed
3	Regorafenib	NCT02195011	Colorectal Neoplasms	Completed
4	FOLFOX6m	NCT01721954	Colorectal Cancer Metastatic	Completed
5	FOLFOX6, Bevacizumab	NCT00735241	Colorectal Carcinoma Liver Metastases	Withdrawn
6	Cetuximab, Irinotecan	NCT00766220	Colon Cancer Colorectal Cancer	Withdrawn
**Nanoparticles**				
7	AGuIX gadolinium-based nanoparticles	NCT04899908	Brain Cancer Brain Metastases Melanoma Lung Cancer Breast Cancer HER2-positive Breast Cancer Colorectal Cancer Gastrointestinal Cancer SRS SRT	Recruiting
8	TKM-080301	NCT01437007	Colorectal Cancer with Hepatic Metastases	Completed
9	Indocyanine green	NCT05092750	Colorectal Cancer	Not yet recruiting
**Liposomes**				
10	Irinotecan HCl Floxuridine	NCT00361842	Colorectal Neoplasms	Completed
11	SN-38 liposome	NCT00311610	Colorectal Cancer	Completed
12	Fluorouracil Irinotecan Sucrosofate Leucovorin Calcium Rucaparib	NCT03337087	Metastatic Colorectal, Carcinoma Stage IVA Colorectal Cancer AJCC v7 Stage IVB Colorectal Cancer AJCC v7	Recruiting
13	Bevacizumab, Fluorouracil, Irinotecan hydrochloride leucovorin calcium irinotecan hydrochloride PEP02 Bevacizumab	NCT01375816	Colorectal Cancer	Terminated
14	Promitil Capecitabine Bevacizumab	NCT01705002	Colorectal Cancer	Completed

**Table 4:** Clinical trial outcomes of enema-based drug delivery for rectal administration.

S. No.	Drug	NCT Number	Condition	Status
1	Lidocaine Hydrochloride	NCT03797703	Hemorrhoids	Completed
2	Pico-Salax, fleet enema	NCT05148494	Colorectal Neoplasms	Recruiting
3	Niclosamide	NCT03521232	Ulcerative Colitis Ulcerative Proctitis Ulcerative Proctosigmoiditis	Recruiting
4	Fleet	NCT02468726	Colorectal Cancer	Completed
5	PUR 0110	NCT01149707	Left-Sided Ulcerative Colitis Proctosigmoiditis	Completed

**Table 5:** Clinical trial outcomes of tablets-based drug delivery for rectal administration.

S. No.	Drug	NCT Number	Condition	Status
1	Imodium	NCT00933465	Fecal Incontinence	Withdrawn
2	Irinotecan	NCT03295084	Metastatic Colorectal Cancer	Completed
3	Capecitabine	NCT01493336	Colorectal Cancer	Completed
4	Diazepam	NCT04216797	Levator Ani Syndrome	Recruiting
5	Regorafenib	NCT03946917	Colorectal Cancer	-
6	Imipramine Hydrochloride	NCT03102645	Fecal Incontinence	Completed
7	AmoxicillinClavulanate	NCT01012843	Anal Fistula	Completed
8	Apatinib Mesylate	NCT03271255	Colorectal Neoplasms Intestinal Neoplasms Gastrointestinal Neoplasms Digestive System Neoplasms	Recruting
9	Metronidazole	NCT04264676	Colorectal Cancer Stage II Colorectal Cancer Stage III	Recruiting
10	Aspirin and Metformin	NCT05158374	Colorectal Cancer Colorectal Neoplasms Colorectal Adenoma	Not yet recruiting
11	Apatinib Mesylate	NCT03743428	Colorectal Neoplasm	Recruiting
12	Niclosamide	NCT02519582	Colorectal Cancer	-
13	Thalidomide	NCT05266820	Metastatic Colorectal Cancer	Recruiting
14	Apatinib	NCT01531777	Colorectal Cancer	Completed
15	Regorafenib	NCT01939223	Colorectal Neoplasms	Terminated
16	Rifaximin	NCT01345175	Rectal cancer	Active
17	Regorafenib	NCT02466009	Metastatic Colorectal Cancer	Completed
18	Selinexor, Pembrolizumab, Trifluridine, Tipiracil	NCT04854434	Metastatic Colorectal Cancer	Active
19	Pembrolizumab, lenvatinib, regorafenib	NCT04776148	Colorectal Neoplasms	Active
20	irinotecan, leucovorin, and 5fluorouracil	NCT00967616	Colorectal Cancer	Completed
21	Regorafenib	NCT01103323	Metastatic Colorectal Cancer	Completed
22	Clindamycin	NCT02585141	Anal Fistulas	Completed
23	Dasatinib, bevacizumab, Oxaliplatin, Capecitabine	NCT00920868	Metastatic Colorectal Cancer	Completed
24	Acetylsalicylic acid	NCT02647099	Colorectal Cancer	Completed
25	Artesunate	NCT02633098	Colorectal Cancer Bowel Cancer	Active
26	Regorafenib	NCT01853319	Colorectal Neoplasms	Completed
27	Capecitabine, Perifosine	NCT01097018	Colorectal Cancer	Completed
28	Dacomitinib, Docetaxel	NCT02039336	Colorectal Cancer	-
29	Lapatinib, trametinib	NCT02230553	Colorectal Cancer	-
30	Cabozantinib, Nivolumab	NCT04963283	Colorectal Adenocarcinoma Colon Cancer Colon Adenocarcinoma Rectum Cancer Rectal Cancer Rectal Adenocarcinoma Colorectal Cancer	Recruiting
31	Tucatinib	NCT05382364	Colorectal Cancer	Recruiting

**Table 6:** Clinically approved rectal formulations for local and systemic action.

S. No.	Drug	Indication	Brand Name	Dosage Form
**For Local Action**				
1	Bisacodyl	Constipation	Dulcolax Bisalax	Suppository Enema
2	Glycerol	Constipation	Glycerol	Suppository
3	Saline laxatives	ConstipationBowel	Micolette Microlax	Enema
4	Mesalazine	Inflammatory bowel disease	Pentasa Salofalk	Suppository Enema Rectal foam
5	Budesonide	Anti-inflammatory	Budenofalk	Rectal foam
6	Prednisolone	Anti-inflammatory	Colifoam	Rectal foam
7	Hydrocortisone	Anti-inflammatory	Predsol Colocort	Suppository Enema
8	Polystyrene sulfonate resins	Hyperkalemia	Resonium A	Enema
9	Glyceryl Trinitrate	Anal fissure, haemorrhoids	Rectogesic	Ointment
**For Systemic Action**				
10	Acetaminophen	Pain, fever	Panadol Acephen Fever All	Suppository
11	Oxycodone	Pain	Proladone	Suppository
12	Ondansetron	Nausea and vomiting	Zofran	Suppository
13	Caffeine + ergotamine	Migraine	Migergot	Suppository
14	Prochlorperazine	Nausea and vomiting	Compro	Suppository
15	Promethazine	Antihistamine	Phenergan	Suppository
16	Ibuprofen	Pain, fever	Nurofen	Suppository
17	Diclofenac	Pain, fever	Voltaren	Suppository
18	Indomethacin	Pain	Indocin	Suppository
19	Diazepam	Seizures, sedation	Diazepam rectal solutionDiastatAcuDial	EnemaGel

**Table 7:** Patented formulations for rectal drug administration.

S. No.	Patent	Title	Claim	Ref.
1	CA2037101C	Omeprazole compositions designed for administration in rectum	A stabilized rectal suppository containing omeprazole as an active ingredient and amino acid as a stabilizer.	[110]
2	US20120237489A1	Suppository for rectal, vaginal, or urethral administration containing a probiotic, an antibiotic, and an unsaturated non-esterified fatty acid	A suppository for rectal, vaginal, or urethral administration comprising at least one probiotic, one antibiotic, and one unsaturated, non-esterified fatty acid.	[111]
3	US8217083B2	Mesalamine suppository	A mesalamine rectal suppository comprising mesalamine and an oily or fatty base treating active ulcerative proctitis in a patient.	[112]
4	US6677319B1	Phosphatidylcholine as medication with protective effect large intestinal mucosa	A method of treating diseases of the colon mucosa, comprising administering a therapeutically effective amount of substrate phosphatidylcholine in a pH-dependent delayed time-release preparation.	[113]
5	WO2011072861A1	Suppository comprising pantoprazole	A suppository comprising at least one pellet and suppository base, wherein the pellet comprises a core and an inert layer surrounding the core, wherein the core comprised pantoprazole.	[114]
6	WO2010143004A2	Glycerol-free osmotic laxative suppository	Osmotic component, stabilizer, and wetting agent containing laxative suppository, wherein the osmotic component and the stabilizer is PEG 200 and/or a polyethylene glycol with higher molecular weight.	[115]
7	EP1492538A1	Meloxicam suppositories containing, e.g., polyethylene glycol	A suppository containing a composition, of meloxicam or a pharmaceutically acceptable excipient, characterized in that at least one of the excipients is a polyalkylene glycol, for the treatment or prevention of polyarthritis, rheumatoid arthritis or inflammation diseases.	[116]
8	US20160002278A1	Pharmaceutical composition for rectal Administration	A pharmaceutical composition for rectal administration in the form of a foam comprising fidaxomicin.	[117]
9	CN1424047A	**A Safety quick effective rectal suppository in glycerin, sodium chloride, and water**	Glycerol, sodium chlorideandwater were used to prepare the present invention and made a kind of liquid laxative with safety, quick onset of action.	[118]
10	WO2008141368A1	**Combination laxative compositions comprising a colonic stimulant and a bulking laxative**	A method of treating constipation in a subject in need thereof comprising the step of providing to said subject a combination of a colonic stimulant and a bulking laxative.	[119]
11	WO2017046343A1	Compositions for rectal administration in the treatment of ulcerative colitis and methods using same	A dry composition for the rectal treatment of IBD comprising an active compound or a pharmaceutically acceptable salt or stereoisomer, an anti-caking agent, and a lubricant, wherein the pH of the dry composition is between about pH 3.0 and about pH 5.0.	[120]

## Data Availability

Not applicable.
